# Evaluation of Dietary Approaches for the Treatment of Non-Alcoholic Fatty Liver Disease: A Systematic Review

**DOI:** 10.3390/nu11123064

**Published:** 2019-12-16

**Authors:** Naba Saeed, Brian Nadeau, Carol Shannon, Monica Tincopa

**Affiliations:** 1 Department of Internal Medicine, Division of Gastroenterology and Hepatology, University of Michigan Health System, Ann Arbor, MI 48109, USA; nasaeed@med.umich.edu (N.S.); brnadeau@med.umich.edu (B.N.); 2 Taubman Health Sciences Library, Ann Arbor, MI 48109, USA; cshannon@umich.edu

**Keywords:** non-alcoholic fatty liver disease, hepatology, nutrition, weight loss

## Abstract

Lifestyle interventions, namely optimizing nutrition and increasing physical activity, remain the cornerstone of therapy for non-alcoholic fatty liver disease (NAFLD), as this can lead to the significant improvement or resolution of disease. The optimal nutritional approach to treat NAFLD remains unclear. The aim of this systematic review is to evaluate the effectiveness of different nutritional patterns on hepatic, metabolic, and weight-loss endpoints. MEDLINE via PubMed, Embase, Scopus, and Google Scholar were searched. Randomized trials of dietary interventions alone for adults with NAFLD were selected. Two authors independently reviewed articles, to select eligible studies, and performed data abstraction. Six studies, representing 317 patients, were included. The participants had a median age of 46, mean body mass index (BMI) 31.5 and were 64.3% male. The mean study duration was 16.33 ± 8.62 weeks. Reduction in hepatic steatosis (HS) was statistically significant in 3/5 Mediterranean Diet (MD), one low-carbohydrate, one intermittent fasting (IF) and 1/2 low fat (LF) diet interventions. A total of 3/5 studies using MD, 1/2 LF interventions, and the one IF intervention demonstrated significant reductions in weight. In conclusion, there appears to be most data in support of MD-based interventions, though further randomized trials are needed to assess comparative effectiveness for NAFLD.

## 1. Introduction

Non-alcoholic fatty liver disease (NAFLD) has become a leading cause of liver disease worldwide, and its progressive form, non-alcoholic steatohepatitis (NASH), is currently the second most common cause for liver transplantation in the United States [1]. It is estimated that 25%–30% of all adults in the United States likely have underlying NAFLD in the setting of the obesity epidemic [2]. Given its complex pathophysiology, there are currently no Food and Drug Administration (FDA)-approved medications for this condition [3]. The mainstay of therapy remains lifestyle modifications, with improvement in nutrition and increase in physical activity targeted toward weight loss [4]. Paired liver biopsy studies have demonstrated that a ≥5% loss of body weight is associated with significant reductions in hepatic steatosis (HS), ≥7% weight loss is associated with reduction in hepatic inflammation, and a ≥10% loss with reduction in fibrosis [5].

Given that nutritional changes account for approximately 80%–90% of weight loss (as opposed to changes in physical activity), there has been an appropriate focus on nutritional patterns associated with the development and progression of NAFLD and NASH [6]. There have been numerous cross-sectional and epidemiological studies that have associated certain nutrients and patterns of dietary intake with NAFLD. There is a paucity of high-quality data that has evaluated the isolated impact of different diets on NAFLD and NASH specifically. This is in large part due to the logistical challenges of conducting randomized controlled trials focused on diets. The available data clearly highlight the correlation with intake of high-fructose corn syrup and red and processed meats, as well as diets high in saturated fat [7,8,9]. As an alternative to content modification, other studies have focused on total caloric restriction, with an estimated reduction in caloric intake by ≥30%, or by 750–1000 Kcal/day, linked with improved insulin resistance (IR) and HS [10,11]. Overall, there remains a lack of consensus regarding the most effective diet for the treatment or prevention of NAFLD. Some argue for carbohydrate-restricted diets, particularly among patients with concurrent diabetes [12]. Others have focused on low-fat regimens, and some on ketogenic diets, though data have emerged that raise concern for increased incidence of NAFLD with ketogenic diets [13,14,15,16]. Recently, there has been a strong focus on use of Mediterranean-based diets, as these have shown promise in reducing the risk of cardiovascular disease [17,18,19]. In this systematic review, we evaluate the effectiveness of different nutritional patterns on hepatic endpoints of interest, including reduction of hepatic steatosis (HS) and fibrosis, weight loss, and metabolic endpoints.

## 2. Materials and Methods

### 2.1. Data Sources and Search Strategy

Following the Preferred Reporting Items for Systematic Reviews and Meta-Analyses (PRISMA) recommendations, we performed serial literature searches for articles of interest, with the help of an expert research librarian [20]. MEDLINE (via PubMed), EMBASE, Scopus, and Google Scholar were searched, using the following keywords: “non-alcoholic fatty liver disease”, “non-alcoholic steatohepatitis”, “NASH”, “NAFLD”, “diet”, and “treatment”. Boolean operators and medical subject heading terms, as well as other controlled vocabulary, were used to enhance electronic searches. The specific search strategy is shown in Table S1.

**Figure 1:**
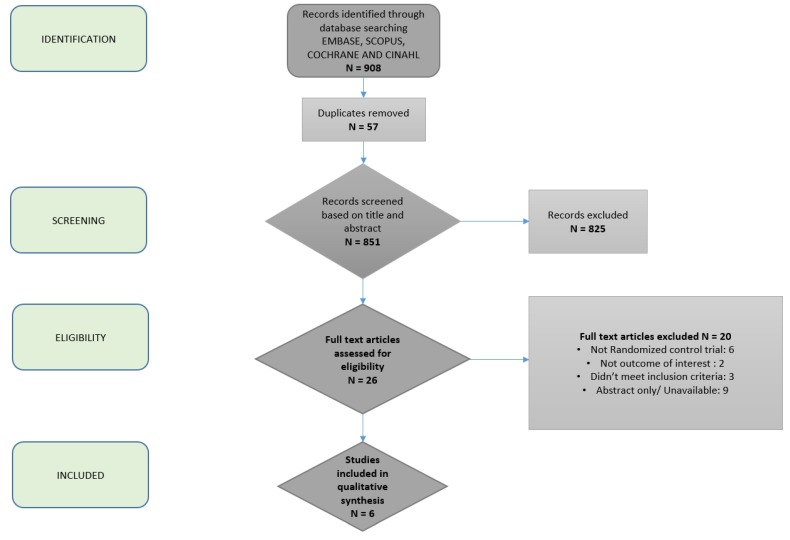
Flowchart depicting article selection process.

All human patient studies published in full text or abstract were eligible for inclusion. Additional studies of interest were identified by searches of bibliographies and consultation with clinical experts on the topic. The search timeframe was selected to begin in 2000 to reflect the current metabolic disease burden and prevalence of obesity. The initial search was performed in April 2019. The search was last updated on 16 November 2019. The process of study inclusion is depicted in Figure 1.

### 2.2. Study Eligibility and Selection Criteria

Two study authors determined study eligibility. Studies were screened for inclusion by N.S. and B.N. Differences in opinion regarding study inclusion were resolved through consensus. Adult (≥18 years) human patient studies that were randomized trials among patients with NAFLD, that compared two or more diets, or one specific diet to usual care, were eligible for inclusion. NAFLD was defined based on liver biopsy or imaging findings of hepatic steatosis without another clear cause (i.e., alcohol). The dietary intervention must have occurred over at least a 4-week period. Diets of interest included the Mediterranean diet (MD), low carbohydrate (LCD), ketogenic diet, low-fat diet (LF), very low-calorie diet (VLCD), intermittent fasting (IF), and DASH diet. When documented, total calorie restriction was also noted. The primary outcome of interest was reduction in hepatic steatosis, based on either imaging or biopsy. Secondary outcomes of interest included changes in hepatic fibrosis (assessed by liver biopsy, advanced imaging or biomarkers (NFS, FIB-4, APRI)), change in liver enzymes, and overall weight loss patterns.

We excluded studies that were case reports, case series, cross-sectional, or case control studies. We also excluded studies that enrolled patients with cirrhosis (as nutritional needs and response to dietary interventions differ in this patient population), animal studies, studies in pediatric populations, those without available full text, and those with no translation in the English language. Importantly, those studies assessing lifestyle modifications in NAFLD/non-alcoholic steatohepatitis (NASH) with an exercise treatment arm, or combined exercise plus diet intervention, were excluded. This was done in order to isolate the specific impact of dietary interventions alone for the treatment of NAFLD. Specific inclusion and exclusion criteria used in each individual study are listed in Table 1.

### 2.3. Data Abstraction and Validity Assessment

Data from eligible studies were abstracted by two authors (N.S. and B.N.), using a standardized template adapted from the Cochrane Collaboration. For all studies, we recorded the following: study design, sample size, patient population characteristics, duration of follow-up, relevant comorbidities, interventions used, method of adherence assessment for intervention, and outcomes measured. We accepted the outcome definitions as stated by each study, without independently validating or reviewing their data.

### 2.4. Assessment of Risk of Bias and Study Quality

Two authors independently assessed the risk of study bias and study quality, using the Downs and Black checklist. This system uses a 27-question scale to assess the quality of a study based on five domains: reporting, external validity, internal validity (bias), internal validity (confounding), and power [21].

### 2.5. Data Synthesis and Analysis

Two authors synthesized the results of the included studies. Studies were categorized according to type of diet evaluated and the outcome of interest assessed. Given the substantial variation in study design across included studies, meta-analysis was not able to be performed.

## 3. Results

### 3.1. Studies Included in the Systematic Review

A total of 908 studies were identified by our expert medical librarian, C.S. After title review by two independent authors, fifty-seven duplicates were removed, leaving 851 unique articles (Figure 1). On the basis of abstract review, 26 were selected for full-text review. Two study authors classified six articles as meeting the predefined criteria for analysis [17,22,23,24,25,26]. A total of 317 patients from six unique populations were represented. Five out of the six studies assessed the effects of the Mediterranean diet (MD), and the remaining study assessed modified alternate-day calorie restriction (MACR, a type of intermittent fasting (IF) diet). Comparison dietary interventions included low fat, low carbohydrate, and usual care. Hepatic steatosis (%) was measured with magnetic resonance spectroscopy (MRS) or ultrasound (US) in 5/6 studies. Liver stiffness was measured using shear wave elastography in 4/6 studies.

### 3.2. Characteristics of Studies

The six studies represented populations from Italy (2/6), Greece, Australia (2/6), and Malaysia. All had relatively small sample sizes, with an approximate mean of 50 (range 12–98). Baseline characteristics are described in Table 1. For purposes of statistical description of age and BMI, the study by Misciagna et al. (*n* = 98) was excluded, as they did not report numerical values; instead, they reported number and percent of patients within certain range categories [22]. Amongst the remaining 219 study participants, the mean age was 46.30 years ± 4.84. Race was only reported by Properzi et al., with 82.4% Caucasians [25]. The mean baseline BMI was 31.50 ± 0.94 kg/m^2^. Of all 317 participants, the majority were male (64.3%), and the mean study duration was 16.33 ± 8.62 weeks (range: six weeks to six months). Three studies excluded diabetics, whilst two studies reported 1/3 and 1/2 of their study participants as being diabetic, and one study did not report patient comorbidities. Detailed inclusion and exclusion criteria for each individual study are reported in Table 1.

### 3.3. Impact of Diets on Hepatic Outcomes

A detailed review of outcomes measured in studies of interest is provided in Table 2. Among studies incorporating an MD, four of the five studies evaluated the impact on HS, and of these, three noted a statistically significant improvement in the amount of HS with the MD intervention [17,22,24,25]. The one study evaluating a low-carbohydrate diet also noted a significant reduction in NAFLD score based on the US [22]. Two study arms employed a low-fat diet, but only one of these arms had a statistically significant reduction in amount of hepatic steatosis [17,25]. The intermittent-fasting arm study also showed statistically significant improvement in amount of hepatic steatosis [26]. Four studies evaluated change in liver stiffness via elastography, with 2/3 MD-based interventions associated with statistically significantly improved liver stiffness measurements, which was also seen in the MACR diet intervention. All six studies evaluated the impact of the dietary intervention on alanine aminotransferase (ALT) levels, but only half of these studies demonstrated a significant improvement (LGMD and INRAN, MD and LF and MACR). Patients achieved weight loss in all of these studies, with significant weight loss in 5/6 studies [22,25,26].

### 3.4. Impact of Diets on Insulin Sensitivity and Lipid Profiles

Again, three studies excluded patients with diabetes, and one excluded patients with a HgA1c > 8.5%. Four studies assessed for the impact on HOMA-IR, and among these, the MD was shown to improve insulin sensitivity in two studies and to have no effect in another two studies [17,23,24,25]. A LF diet was shown to significantly improve HOMA-IR in one study, but did not have a significant impact in a second study [17,25]. Changes in triglycerides (TG) were evaluated in all six studies, with two of five studies evaluating MD showing statistically significant improvement in TG [24,25].

### 3.5. Impact of Diets on Weight Loss

Amongst the studies employing an MD, three demonstrated at statistically significant reduction in weight and body mass index (BMI) [23,24,25]. Of the two studies that applied a LF intervention arm, one cohort achieved significant weight loss, whereas the second did not [17,25]. The one study using MACR also reported a significant decrease in weight. [26].

### 3.6. Quality Assessment and Risk of Bias

Overall, the studies were assessed as being of moderate quality (Table S2). Four studies had patient cohorts with limited representativeness compared to the overall patient population of interest, and four studies were considered to be of low power.

## 4. Discussion

Obesity and its numerous metabolic comorbidities have become public health crises in recent years [27]. At the crux of this issue is the pervasiveness of nutrient-poor diets, combined with highly sedentary lifestyles that predispose individuals to incident and prevalent metabolic disease. In this setting, NAFLD has emerged as a leading cause of chronic liver disease worldwide. If left unchecked, it will have demands that far out-supply our health-care provider and transplant-organ availability needs. The effectiveness of current experimental agents to reverse hepatocyte damage remains unknown [28]. It is likely that, without lifestyle changes, these medications will be a lifelong requirement, creating significant cost and potential for adverse drug effects. Numerous studies have clearly shown that HS, steatohepatitis and hepatic fibrosis associated with NAFLD and NASH can improve or resolve with sustained weight loss. The optimal nutritional approach to achieve weight loss in this patient population remains unknown, however; in large part this is due to the difficulties of conducting high-quality randomized trials focused on nutrition. In this systematic review, we highlighted the available data, to identify specific benefits of commonly recommended nutritional programs for patients with NAFLD.

In this context, our review details several areas in need of attention from the hepatology community, in order to be able to provide evidence-based guidance to our patients. Firstly, only six studies met the inclusion criteria for this review, highlighting the significant knowledge gap that needs to be addressed in order to objectively compare the effectiveness of different dietary programs for the treatment of NAFLD. Among these six studies, the vast majority evaluated an MD. While this adds to our knowledge on the multisystem benefits of am MD diet, little is known about commonly used diets, like low-carbohydrate, low-fat, and the recently popular ketogenic and intermittent fasting diets, for patients with NAFLD. Secondly, there remains an important need to employ a standardized approach to studying these types of lifestyle interventions in this patient population, as the notable heterogeneity in study design precludes meta-analysis and makes it challenging to comparatively assess interventions. Of note, for these included studies, three studies excluded patients with diabetes, and one excluded patients with poorly controlled diabetes, which significantly reduced the ability to translate the findings of these studies to the larger patient population. The duration of the intervention and relevant comparison group should also be more routinely applied. Ideally, six-month-based interventions, with standard of care control groups would provide meaningful data that, would speak to feasibility, sustainability, and comparative effectiveness to usual care.

Despite the numerous limitations in the existing data, the available results reinforce the effectiveness of nutrition-based lifestyle programs for the treatment and resolution of NAFLD. In particular, MD-based interventions appear to be very effective for reducing hepatic steatosis. MDs were also shown to be associated with significant weight loss in the majority of studies, as well as improvement in TG in some, but not all, MD-based interventions. Though not much can be stated about the impact of low-carbohydrate or low-fat diets, due to the limited number of interventions that met inclusion criteria with these approaches, the hepatic and metabolic outcomes seen with these interventions appeared less robust than those seen with the MD-based interventions. Lastly, intermittent fasting was associated with improvements in HS and weight, but was only evaluated in one study.

As is the case with any nutrition-based intervention, there remain concerns about monitoring adherence and sustainability of any type of intervention. Many nutrition and behavioral modification experts emphasize the importance of tailoring dietary recommendations to individual patients in order to optimize feasibility and therefore sustainability and efficacy of these nutritional plans. As hypothesized in the literature, we suspect that patient preference and ability to remain adherent to dietary regimens (as well as differences in acceptable adherence thresholds amongst our studies) likely account for the conflicting data in terms of efficacy of different types of diets for NAFLD.

Consequently, more rigorous, randomized controlled trials focused exclusively on NAFLD patients, investigating the most relevant dietary programs (including low-carbohydrate and MD), are needed.

## Figures and Tables

**Table 1:** Key characteristics of included articles.

Author, Year Country	Study Design Duration	N	Demographics Mean Age (Years), Sex	Baseline Mean BMI (kg/m^2^) Comorbidities	Inclusion Criteria	Exclusion Criteria
Ryan, 2013 Australia	Randomized, crossover 6 weeks	12 (each was their own control)	Age: 55 ± 14 Sex: 50% female	32.0 ± 4	Biopsy-proven NAFLD MetSyn <7 alcoholic drinks/week for women and <10/week for men	Diabetes
Misciagna, 2017 Italy	Double-blind, randomized controlled trial 6 months	98 LGMID: 50 Control: 48	Age: 18–79 Sex: 26.5% female	Normal (*n* = 3 in LGMID) Overweight (*n* = 13 in both groups) Obese (*n* = 35 INRAN group, *n* = 34 LGIMD group)	Moderate to severe NAFLD based on US	Overt CVD or revascularization Stroke Clinical PAD Diabetes Severe medical condition that may impair participation Recent weight loss On special diet or weight-loss program Inability to follow MD
Katsagoni, 2018 Greece	Single-blind, randomized, controlled 6 months	63 CG: 21 recruited, 7 dropped out MDG: 21 MLG: 21	Age: 18–65 Sex: 31.7 % female	25–40	US and/or biopsy-proven NAFLD and elevated ALT and/or У-GGT	Other causes of liver disease or steatosis Weekly alcohol consumption of >210 g for men or >140 g for women -Diabetes Currently on weight-loss diet
Abenavoli, 2017 Italy	Randomized controlled 6 months	50 MD: 20 MD + AO: 20 CG: 10	Age: MD: 52 (40–60) MD+AO: 46 (40–57) Control:33 (28–40) Sex (% female): MD: 40% MD+AO: 20% Control: 40%	MD: 31 (29–33) MD+AO: 29 (28–32) Control: 29 (27–31)	Adults with NAFLD BMI > 25 kg/m^2^	Hepatitis B/C Other systemic diseases (cardiac, renal, autoimmune, metabolic) Treatment with insulin Smoking Significant alcohol use Recreational drug use Exposure to liver toxins
Properzi, 2018 Australia	Single-blinded, randomized controlled 12 weeks	51 MD: 26 LF: 25	Age: MD: 51 + 13.36 LF: 53 + 9.06 Race: MD: 80.8% white LD: 84% white Sex: MD: 42% female LF: 56% female	MD: 31.5 ± 4.1 LF: 30.2 ± 5.6 For MD, LF respectively: DM (30%, 28%) HTN (34.6%, 40%) HLD (57.7%, 44%) CVD (19.2%, 12%)	NAFLD diagnosis with HS > 5.5% on MRS	Unstable body weight variation (>5% change in prior 3 months) Use of weight loss medications HbA1c > 8.5% Pioglitazone use Decompensated cirrhosis Renal failure Malignancy Atrial fibrillation Pregnancy or lactation Current smoking Significant alcohol use
Johari, 2019 Malaysia	Randomized, controlled 8 weeks	43 MACR: 33 Control: 10	Age: 45.33 ± 10.77 Sex: 23.2% female	31.60 ± 5.19 17/33 with diabetes (equal proportions in intervention and control arms)	Ages 18–70 years BMI 17.5–40 kg/m^2^ Elevated ALT and AST	Other cause of liver disease Significant alcohol intake Pregnancy Active weight loss program/weight loss Medication use Substance abuse Psychiatric disease Unable to tolerate fasting

**Table 2:** Assessments and outcomes.

Author	Dietary Intervention	Adherence Assessment	Baseline Steatosis/Fibrosis	Change in Hepatic Steatosis/Fibro-sis	Changes in Body Weight (kg) or BMI (kg/m^2^) (Means)	Change in AST and/or ALT (IU/L)	Change in Total Cholesterol and Triglycerides (md/dl)	Change in Insulin Sensitivity (HOMA-IR) (Normal < 2)
Ryan	6 weeks MD (40% mon-and omega-3 polyunsaturated fat, 40% carbohydrate, 20% protein) with 6-week washout period during crossover, followed by 6 weeks LF/HCD (30% fat, 50% carbohydrate, 20% protein); food was supplied; up to 2 alcoholic drinks 5 days per week	7-day food diary at the beginning and end of dietary intervention	IHL% Based on H-MRS: MD: 14.2 ± 11.7 LF/HCD: 11.2 ± 4.4	IHL% Based on H-MRS: MD: 8.6 ± 7.0 (*p* < 0.05) LF/HCD: 10.0 ± 3.6 (*p* > 0.05)	MD: Wt: 88.3 → 87.3 (*p* > 0.05) BMI: 31.5 → 31.2 (*p* > 0.05) LF/HCD: Wt: 90.7 → 80.3 (*p* > 0.05) BMI: 31.5 → 30.8 (*p* > 0.05)	No significant change in ALT with either diet	MD: TG: 224 → 201 (*p* > 0.05) LF/HCD: TG: 222 → 221 (*p* > 0.05)	MD: 4.7 → 3.0 (*p* < 0.01) LF/HCD: 4.1 → 3.9 (*p* > 0.05)
Misciagna *	LGIMD (*n* = 50) or INRAN (*n* = 48)	MAI based on weekly for the first month then monthly diet journal entries	Moderate to severe NAFLD based on US Moderate NAFLD: *n* = 34 in INRAN, *n* = 35 in LGIMD Severe NAFLD: *n* = 14 in INRAN, *n* = 15 in LGIMD	Significant reduction in NAFLD score based on US in both men and women in the LGIMD group until 55 years of age	Reduction in the number of obese patients in both diet groups at 6 months, but increase in number of overweight patients in both groups	Significant reduction in ALT in both groups AST normal at baseline and end of study in all patients in both groups	LGIMD: Reported as number of people with improvement TG: 11% patients levels normalized	
Katsa-goni	CG or MDG, or MLG; All 3 groups given energy-restriction regimen, with 45% carbohydrates, 20% protein, and 35% lipids CG: given general written dietary guidelines for healthy lifestyle MDG and MLG: 7 60-min small group dietary counseling sessions to enhance MD adherence (every 2 weeks, for first 2 months, then monthly for next 4 months) MLG: given goal of moderate-to-vigorous physical activity ≥30 min/day and 7–9 h of sleep per day	-Self-monitoring: Special forms for intervention goals or 3-day dietary records, Med. Diet Score, 69-itm FFQ and 24 h food recall at baseline and end of study MLG group given a pedometer with goal of 10,000 steps/day	MDG: TE: 6.6 kPa NFS: −2.36 MLG: TE: 7.1 kPa NFS: −2.11	MDG: TE: 6.1 kPa (*p* = 0.002) NFS: -2.38 (*p* = 0.65) MLG: TE: 6.1 kPa (*p* = 0.002) NFS: −2.09 (*p* = 0.65)	MDG Wt loss: −5.4% loss (*p* = 0.01) BMI: 31.6 → 28.2 (*p* = 0.008) MLG: Wt loss: −6.3% (*p*= 0.01) BMI: 32.44 → 30.55 (*p* = 0.008)	MDG: ALT 51 → 34 (*p* = 0.09) MLG: ALT 54 → 32(*p* = 0.09) Per protocol: Non-significant reduction in ALT in MDG	MDG: TC: 197.2 → 185.6 (*p* = 0.08) TG: 132.86 → 106.29 (*p* = 0.28)	MDG: 3.4 → 2.6 (*p* = 0.60)
Abena-voli	MD vs. MD + Antioxidant (AO) vs. control (regular diet)	Monthly phone calls	MD: FL index: 71 (56–85) TE: 8.1 (6.7–9.2) kPa MD + AO: FL Index: 58 (42–69) TE: 6.9 (6.7–7.2) kPa	MD: FL index: 45 (39–69), (*p* = 0.002) TE: 6.0 (5.1–7.0) kPa (*p* = 0.0001) MD + AO: FL Index: 38 (29–45) (*p* = 0.003) TE: 5.0 (4.7–5.2) kPa (*p* = 0.0001)	MD: Wt: 83 → 78 (*p* = 0.0001) BMI: 31 → 29 (*p* = 0.0001) MD + AO Wt: 90 → 81 (*p* = 0.002) BMI: 29 → 27 (*p* = 0.0001)	No significant change in either group	MD: TC: 189 → 156 (*p* = 0.0001) TG: 140 → 85 (*p* = 0.0001) MD+AO: TC: 198 → 152 (*p* = 0.0001) TG: 106 → 75 (*p* = 0.011)	MD: 1.9 (0.9–2.4) → 1.8 (0.6–3.4) (*p* = 0.985) MD + AO: 4 (3–6) → 2 (1–2) (*p* = 0.001)
Properzi	MD (40% carbs, 40% fats, and 20% protein) vs. LF diet (50% carbs, 30% fat, and 20% protein)	Weekly dietician follow-up x 4 weeks, then monthly dietician follow-up	MD: Hepatic fat on MRS 34.2 ± 16.3% TE: 12.4 ± 15.4 kPa LF: Hepatic fat on MRS 21.5 ± 10%, TE: 7.0 ± 3.8 kPa	MD: Hepatic fat on MRS 24.0 ± 14.7% (*p* < 0.001) Liver stiffness: 11.7 ± 15.3 kPa (*p* = 0.11) LF: Hepatic fat on MRS 15.3 ± 7.7% (*p* < 0.001), Liver stiffness: 7.0 ± 6.0 kPa (*p* = 0.20)	MD: Wt: 89.3 → 87.3 (*p* < 0.001) BMI: 31.8 (4.0) → 31.1 (4.0) (*p* < 0.001) LF: Wt: 81.3 → 79.6 (*p* = 0.001) BMI: 30.1(5.69) → 29.5 (5.8) (*p* = 0.001)	MD: ALT 77 → 69 (*p* = 0.049) LF: ALT 68 → 56 (*p* = 0.004)	MD: TC: 184.8 → 175.2 (*p* = 0.010) TG: 165.6 → 144.2 (*p* = 0.008) LF: TC: 202.2 → 199.2 (*p* = 0.27) TG:144.4 → 139.9 (*p* = 0.38)	MD: 3.91 → 3.63 (*p* = 0.263) LF: 2.76 → 2.95 (*p* = 0.040)
Johari	MACR (type of IF) 70% calorie restricted diet between 2:00 p.m. and 8:00 p.m. one day, alternating with regular diet next day	Intermittent phone calls to patients by investigator Biweekly follow-up with dietician	Steatosis on US: 1.93 TE 5.87 kPA	Steatosis on US: 1.43 (Δ = 0.50, *p* = 0.001) Elastography: 5.01 kPA (Δ = 0.86 kPA, *p* = 0.001)	Weight 80.8 → 78.9 (*p* = 0.003) BMI = 31.73 → 30.95 (*p* = 0.03)	ALT 84.3→ 59.17 iU/L (*p* = 0.001) AST 51.4 → 42.7 iU/L (*p* = 0.004)	MACR: TC: 205.72 → 204.18 (*p* = 0.78) TG: 174.5 →185.12 (*p* = 0.58)	

Notes: NR: not reported; LCKD: low-carbohydrate ketogenic diet; MetSy: metabolic syndrome; MD: Mediterranean diet; LF/HCD: low-fat high-carbohydrate. diet; 1H-MRS: magnetic resonance 1H spectroscopy; AST: aspartate aminotransferase; ALT: alanine aminotransferase; NAFLD: non-alcoholic fatty liver disease; SKMD: Spanish ketogenic Mediterranean diet; US: ultrasound; LGIMD, low-glycemic-index Mediterranean diet; INRAN, Italian National Research Institute for Foods and Nutrition; MLG, Mediterranean lifestyle group; CVD, cardiovascular disease; PAD, peripheral artery disease; T2DM, type 2 diabetes mellitus; LCD: low-calorie diet; MACR: modified alternate-day calorie restriction; IF: intermittent fasting; MAI, Mediterranean adequacy index; MD: Mediterranean Diet; MDG, Mediterranean diet group; CG, control group; FFQ, food frequency questionnaire; NFS, NAFLD fibrosis score; ITT, intention-to-treat; NR, not reported; PAL, physical activity level; APAQ, Athens Physical Activity Questionnaire; MET, metabolic units; FL Index: fatty liver index; TE: transient elastography; MRS: magnetic resonance spectroscopy; HbA1c: hemoglobin A1c level; TC: total cholesterol; TG: triglycerides. * Numbers reported in this study were % patients with normal vs. abnormal results, not the actual values.

## Supplementary Materials

The following are available online at https://www.mdpi.com/2072-6643/11/12/3064/s1. Table S1. Search Strategy; Table S2. Downs and Black Checklist.
