# Supplementary material for: Evaluation of Dietary Approaches for the Treatment of Non-Alcoholic Fatty Liver Disease: A Systematic Review
Source: Nutrients. 2019 Dec 16;11(12):3064. doi: 10.3390/nu11123064 (PMC6950283; doi:10.3390/nu11123064)
Supplement: Supplementary file 1 [file nutrients-11-03064-s001.pdf]

Nutrients **2019**, *11*, 3064; doi:10.3390/nu11123064

TABLE S1: Search Strategy

Pubmed	(fatty liver[mh] OR "fatty liver"[tiab] OR Non-alcoholic Fatty Liver Disease[tiab] OR nonalcoholic fatty liver disease[tiab] OR NASH[tiab] OR NAFLD[tiab]) AND (Caloric restriction[mh] OR ketogenic diet[tiab] OR carbohydrate-restricted diet[tiab] OR Mediterranean diet[mh] OR Mediterranean diet[tiab] OR Diet, Fat-restricted[mh] OR Diet, Paleolithic[mh] OR paleolithic diet[tiab] OR Diet, Reducing[mh] OR diet therapy[mh] OR very low calorie diet[tiab] OR DASH diet[tiab] OR intermittent fasting[tiab] OR weight loss[tiab] OR weight loss[mh] OR caloric restriction[tiab])) AND adult[MeSH])) NOT (((fatty liver[mh] OR "fatty liver"[tiab] OR Non-alcoholic Fatty Liver Disease[tiab] OR nonalcoholic fatty liver disease[tiab] OR NASH[tiab] OR NAFLD[tiab]) AND (Caloric restriction[mh] OR ketogenic diet[tiab] OR carbohydrate-restricted diet[tiab] OR Mediterranean diet[mh] OR Mediterranean diet[tiab] OR Diet, Fat-restricted[mh] OR Diet, Paleolithic[mh] OR paleolithic diet[tiab] OR Diet, Reducing[mh] OR diet therapy[mh] OR very low calorie diet[tiab] OR DASH diet[tiab] OR intermittent fasting[tiab] OR weight loss[tiab] OR weight loss[mh] OR caloric restriction[tiab])) AND ((Case Reports[ptyp] OR Review[ptyp]) AND adult[MeSH])) Filters: Publication date from 2000/01/01 to 2019/12/31; English; Adult: 19+ years
Embase	1. 'fatty liver'/exp OR 'nonalcoholic fatty liver'/exp<br>2. 'hepatic steatosis':ab,ti OR 'nonalcoholic fatty liver':ab,ti OR nash:ab,ti OR nafld:ab,ti<br>3. #1 OR #2<br>4. diet therapy'/exp OR 'low carbohydrate diet'/exp OR 'low calorie diet'/exp OR 'mediterranean diet'/exp OR 'diet restriction'/exp OR 'carbohydrate diet'/exp OR 'low fat diet'/exp OR 'body weight loss'/exp N = 506,700<br>5. 'low fat-high carbohydrate diet':ab,ti OR 'ketogenic diet':ab,ti OR 'mediterranean diet':ab,ti OR 'paleolithic diet':ab,ti OR 'reducing diet':ab,ti OR 'weight loss':ab,ti<br>6. #4 OR #5<br>7. #4 AND #6<br>8. #7 AND 'case report'/de<br>9. #7 AND 'review'/it<br>10. #8 OR #9<br>11. #7 NOT #10<br>12. #7 NOT #10 AND ([adult]/lim OR [aged]/lim) AND [2000-2019]/py AND [english]/lim

Scopus Cited Reference Search	Ad Libitum Mediterranean and Low-Fat Diets Both Significantly Reduce Hepatic Steatosis: A Randomized Controlled Trial.<br><br>2. Barriers and Facilitators to Mediterranean Diet Adoption by Patients with Non-alcoholic Fatty Liver Disease in Northern Europe. Not in Embase or Scopus<br><br>3. The Mediterranean diet improves hepatic steatosis and insulin sensitivity in individuals with non-alcoholic fatty liver disease<br><br>4. Effect of Mediterranean Diet and Antioxidant Formulation in Non-Alcoholic Fatty Liver Disease: A Randomized Study.<br><br>5. Effects of a Low-Calorie, Low-Carbohydrate Soy Containing Diet on Systemic Inflammation Among Patients with Nonalcoholic Fatty Liver Disease: A Parallel Randomized Clinical Trial.<br><br>•<br><br>6. Effects of a Low-Calorie, Low-Carbohydrate Soy Containing Diet on Systemic Inflammation Among Patients with Nonalcoholic Fatty Liver Disease: a randomized clinical trial.<br><br>7. Effect of a Low Glycemic Index Mediterranean Diet on Non-Alcoholic Fatty Liver Disease. A Randomized Controlled Clinici Trial.

Nutrients **2019**, *11*, 3064; doi:10.3390/nu11123064

Supplement Table 2: Downs and Black Checklist

**Study Title**	**Reporting (0-10)**	**External Validity (0-3)**	**Internal Validity – Bias (0-7)**	**Internal Validity – Confounding (Selection Bias) (0-6)**	**Power (0-1)**	**Total (0-28)**
**Ryan 2013**	9	0	5	4	0	18
**Misciagna 2017**	8	3	5	5	1	22
**Katsagoni 2018**	10	3	5	5	0	23
**Abenavoli 2017**	7	1	5	4	0	17
**Properzi 2018**	9	1	6	6	0	22
**Johari 2019**	9	1	5	5	1	21
